# Phylotranscriptomics and evolution of key genes for terpene biosynthesis in Pinaceae

**DOI:** 10.3389/fpls.2023.1114579

**Published:** 2023-02-17

**Authors:** Kaibin Jiang, Chengju Du, Linwang Huang, Jiexian Luo, Tianyi Liu, Shaowei Huang

**Affiliations:** ^1^ College of Forestry and Landscape Architecture, South China Agricultural University, Guangzhou, China; ^2^ Guangdong Key Laboratory for Innovative Development and Utilization of Forest Plant Germplasm, South China Agricultural University, Guangzhou, China

**Keywords:** evolution, Pinaceae, phylogeny, P450, species tree, terpene synthase

## Abstract

Pinaceae is the largest family of conifers, dominating forest ecosystems and serving as the backbone of northern, temperate and mountain forests. The terpenoid metabolism of conifers is responsive to pests, diseases, and environmental stress. Determining the phylogeny and evolution of terpene synthase genes in Pinaceae may shed light on early adaptive evolution. We used different inference methods and datasets to reconstruct the Pinaceae phylogeny based on our assembled transcriptomes. We identified the final species tree of Pinaceae by comparing and summarizing different phylogenetic trees. The genes encoding terpene synthase (TPS) and cytochrome P450 proteins in Pinaceae showed a trend of expansion compared with those in Cycas. Gene family analysis revealed that the number of TPS genes decreased while the number of P450 genes increased in loblolly pine. Expression profiles showed that TPSs and P450s were mainly expressed in leaf buds and needles, which may be the result of long-term evolution to protect these two vulnerable tissues. Our research provides insights into the phylogeny and evolution of terpene synthase genes in Pinaceae and offers some useful references for the investigation of terpenoids in conifers.

## Introduction

Gymnosperms are the main components of forest ecosystems, especially in boreal, temperate and montane forests (Sederoff, 2013). Among them, 625 species of conifers are the major parts of forest ecosystems, accounting for 39% of the world’s forests (Jin et al., 2021). Pinaceae is the largest family of conifers and the largest family of gymnosperms (De La Torre et al., 2014), including 11 genera: *Abies* (approximately 50 species), *Cathaya* (1 species), *Cedrus* (4 species), *Keteleeria* (approximately 11 species), *Larix* (approximately 13 species), *Picea* (approximately 50 species), *Pinus* (approximately 80 species), *Pseudolarix* (1 species), *Pseudotsuga* (approximately 18 species), Nothotsuga (1 species), and *Tsuga* (approximately 14 species) (Ran et al., 2018).

Previously, Pinaceae was divided into three subfamilies, Abies, Larix, and Pinaceae, according to their morphological characteristics (Krussmann et al., 1985). Subsequently, on the basis of multiple traits, such as morphological, anatomical, and immunological features, it was considered more reasonable to divide Pinaceae into two large groups (Price et al., 1987; Price et al., 1998). The division of Pinaceae into two clades is supported by molecular phylogenetic studies (Lin et al., 2010; Sudianto et al., 2016), but the phylogenetic positions of some of these genera, such as *Pinus*, *Picea*, and *Cathaya*, are controversial. Phylogenetic studies based on a few genes suggest that *Pinus* is a sister clade to *Picea* or *Cathaya* (Lu et al., 2014; Gernandt et al., 2016) or that the three closely related genera *Pinus*, *Picea* and *Cathaya* should be treated as a trichotomy (Nkongolo et al., 2012). Phylogenetic reconstruction using a small number of genes is susceptible to random errors (Hedtke et al., 2006; Zhang et al., 2015). Transcriptomes and genomes are widely used in phylogenetic and evolutionary studies of plants (Jackson et al., 2022) and animals (Bi et al., 2021; Meyer et al., 2021). However, due to the giga-genome of pine trees, it is difficult to assemble genomes and use them to study evolution in Pinaceae.

Recently, some studies have used complete plastid Genome (Sudianto et al., 2016) or transcriptomes (Ran et al., 2018) to reconstruct the Pinaceae phylogeny. However, they did not systematically compare and summarize different inference methods for species trees. Here, we assembled the transcriptomes of 18 species. Then, we used concatenated and coalescent species tree inference methods as well as different datasets to analyze and further understand the phylogeny of Pinaceae.

On the other hand, conifer stems and leaves produce and release oleoresin, which is an induced defense response against assaults by herbivores, insects, and diseases (Liu et al., 2021). Oleoresin comprises a range of terpenoid chemicals (Keeling and Bohlmann, 2006). The cytosolic mevalonate (MEV) and chloroplast methyl erythritol phosphate (MEP) pathways are the sources of all terpenoid metabolic products in conifers (Celedon and Bohlmann, 2019). The last two stages of resin terpene biosynthesis are controlled by the catalytic enzyme genes TPSs and the P450 superfamily in these two pathways (Niu et al., 2022). Therefore, we examined expansion and contraction of gene families, especially the terpene synthase (TPS) and P450 families of key rate-limiting enzymes for terpene biosynthesis, in different species of Pinaceae.

## Materials and methods

### RNA extraction and sequencing

Whole young leaves were collected from *Pinus taeda*, *Pinus elliottii*, and *Pinus massoniana* at the Forestry Science Institute of Yingde (Guangdong, China) and the Hongling seed orchard of Taishan (Guangdong, China). Collected leaves were stored at -20 °C until RNA extraction. Three 8-year-old *P. taeda* trees, with a similar growth state and free of pests and diseases were selected for sampling. The leaf buds, needles, twigs, trunk phloem, and roots were collected separately from each tree at the same time during the blooming period. Total RNA was extracted from different tissues of *Pinus taeda* using the TIANGEN RNAsecure Plant Kit (Beijing, China) following the manufacturer’s instructions. Sequencing libraries were prepared with insert sizes of 200 bp and sequenced using an Illumina HiSeq 4000 platform.

### Transcriptome sequencing data

From open-access databases, raw transcriptome data for 15 species were downloaded. Among the species, 12 species belong to 10 genera of Pinaceae, including *Abies firma*, *Cathaya argyrophylla*, *Cedrus deodara*, *Keteleeria evelyniana*, *Larix gmelinii*, *Picea abies*, *Picea smithiana*, *Pinus armandii*, *Pinus elliottii*, *Pinus massoniana*, *Pinus taeda*, *Pseudolarix amabilis*, *Pseudotsuga menziesii*, *Tsuga dumosa* and *Tsuga longibracteata*, and the three species *Cycas panzhihuaensis*, *Araucaria cunninghamii*, and *Platycladus orientalis* were used as outgroups (**Supplementary Table S1**).

### Transcriptome assembly and annotation

Individual sequence quality was checked using FastQC, and sequence reports were combined using MultiQC (Ewels et al., 2016). RCORRECTOR v1.0.4 (Song and Florea, 2015) was used to error-correct Illumina RNA-seq reads of the transcriptome sequencing data with default settings. TRIMMOMATIC v0.39 (Bolger et al., 2014) was then used to remove low-quality sequences and adapters with the parameters ‘PE -phred33 ILLUMINACLIP : TruSeq3-PE.fa:2:30:10 LEADING:3 TRAILING:3 SLIDING WINDOW:4:15 MINLEN:80’. TRINITY v2.1.1 (Haas et al., 2013) was used to assemble transcripts with clean and corrected reads. For the transcripts, DIAMOND v0.9.25 (Buchfink et al., 2015) was used to search the protein database (ftp://ftp.uniprot.org/pub/databases/uniprot/current_release/knowledgebase/complete/uniprot_sprot.fasta.gz) for homology support with an E-value of 1E-5. TRANSDECODER v5.5.0 (https://github.com/TransDecoder/TransDecoder/releases) was used to translate the longest transcripts and find candidate coding sequences. Benchmarking Universal Single-Copy Orthologs (BUSCO v5.3.2) (Simao et al., 2015) with the embryophyta_odb10 database (https://busco-data.ezlab.org/v5/data/lineages/arthropoda_odb10.2020-09-10.tar.gz) was used to evaluate annotation completeness and assembly quality in transcriptome and protein modes, respectively.

### Phylogenetic reconstruction

OrthoFinder Version 2.5.4 (Emms and Kelly, 2019) was employed to construct the orthogroups for the transcriptomes with default settings. We gathered three independent datasets to reconstruct the phylogeny of Pinaceae genera: 1) a dataset of 319 single-copy orthologous genes (SCOGs) generated from 15 Pinaceae plant transcriptomes; 2) a dataset of 120 SCOGs of 16 taxa, including 15 Pinaceae species and one Cycadaceae species (outgroup); 3) and another dataset of 54 SCOGs derived from 18 taxa, including 15 Pinaceae plants and three outgroups (an Araucariaceae plant, a Cupressaceae plant and a Cycadaceae plant). TranslatorX (Abascal et al., 2010) was used for multiple gene alignments based on codon (nt), codon 1st+2nd (nt12) and amino acid (aa) sequences with the local version (command: perl translatorx_vLocal.pl -i gene.fa -o gene.out -p M -t F -w 1 -c 1 -g “-b2 = 0.75 -b3 = 8 -b4 = 5 -b5=h -b6=y”). The maximum likelihood (ML) approach was used to build a concatenated tree for the different sequences of each dataset using IQ-TREE (Nguyen et al., 2015). ASTRAL (Zhang et al., 2018) was used to derive a coalescent tree for the different sequences of each dataset.

#### Concatenated phylogenetic tree

With an initial partition scheme of codon locations, including ModelFinder, tree search, and ultrafast bootstrap, IQ-TREE 2 (Minh et al., 2020) was utilized to infer the ML trees. First, the nt, nt12 and aa sequences of orthologous genes were concatenated into supergenes. The auto-best nucleotide substitution models were identified using ModelFinder. IQ-TREE 2 was then used to infer ML trees with the best substitution model. Each ML analysis was performed with 1000 ultrafast bootstrap replicates (-bb 1000).

#### Coalescence-based phylogenetic tree

First, IQ-TREE was used to construct the ML trees based on nt, nt12 and aa sequences of each orthologous gene. ASTRAL was then used to infer the species tree with all ML trees from nt, nt12 or aa sequences. The coalescent species tree with quartet support was summarized using ASTRAL with the default options (-t 8). The topology of the coalescent species tree was produced by ASTRAL using the quartet trees of the ML phylogenies of each gene, which returned quartet scores and posterior probabilities. The gene tree conflicts on the species tree topology were inferred and shown using the PHYPARTS program (Smith et al., 2015) with default parameters. The gene tree discordance and conflicts between various analytical methods and datasets were interpretably visualized and summarized by DISCOVISTA (Sayyari et al., 2018).

### Divergence time estimation

The MCMCTree module of the PAML program (Yang, 2007) was used to calculate divergence times for 16 species based on the nt12 sequences of 120 SCOGs and 2 fossil calibration points from crown group of *Pinus massoniana* (Jin et al., 2021) and *Cycas panzhihuaensis* (Liu et al., 2022) (**Supplementary Table S2**). The dated phylogeny was visualized and annotated using the R package GGTREE (Yu et al., 2017).

### Gene family expansion and contraction analysis

CAFE v4.2.1 software (Han et al., 2013) for computational analysis of gene family evolution was used to analyze variation in gene family size with the following parameters: -p 0.05 -k 4. The Monte Carlo resampling procedure was used to determine the significance of the expansion and contraction of gene families. The script CAFE_fig.py (https://github.com/LKremer/CAFE_fig) was used to summarize and visualize the numbers of expanded and contracted gene families. The online version of the EGGNOG-MAPPER 5.0 database (Huerta-Cepas et al., 2019) (http://eggnog-mapper.embl.de/) was used for gene ontology (GO) annotation of expanded and contracted gene families. The online version of KEGG Automatic Annotation Server (KASS) (Moriya et al., 2007) (https://www.genome.jp/tools/kaas/) was used KEGG Orthology (KO) annotation and KEGG pathway analysis of expanded and contracted gene families. A genomic approach would have been better, but it is difficult to assemble Pinaceae genomes and use them to study gene family.

### Evolution of the P450 family and TPS family

A total of 111 terpene synthases (TPSs) and 23 P450 family proteins from conifer genes (Celedon and Bohlmann, 2019) were used as references to screen homologs in 15 transcriptomes of Pinaceae species, the outgroup (*Cycas panzhihuaensis*), and loblolly pine (*Pinus taeda*) expanded and contracted gene families using BLASTP v2.2.31 with an e-value of 1.0×e-20. CD-Search (Lu et al., 2020) (https://www.ncbi.nlm.nih.gov/Structure/bwrpsb/bwrpsb.cgi) was used to check the conserved domains of all proteins. TBtools (Chen et al., 2020) was used to compare gene structures for each family. Muscle v3.8 (Edgar, 2004) was used for multiple sequence alignments of each family. MEGA7 (Kumar et al., 2016) was then used to build ML-based phylogenetic trees with 500 bootstrap replicates. The trees were visualized with iTOL (Letunic and Bork, 2019) (https://itol.embl.de/itol.cgi).

### Expression patterns of TPS and P450 genes in *pinus taeda*

RNA sequencing reads from different tissues were trimmed using Trimmomatic (Bolger et al., 2014) program and mapped against the assembled *Pinus taeda* transcriptome using bowtie2 by retaining the best alignments. TPM were calculated using the eXpress program, which was incorporated in the Trinity (Haas et al., 2013) package.

## Results

### Completeness of the transcriptome assembly

In the assembly results from Trinity (Haas et al., 2013), the gene numbers of the species ranged from 53,856 to 85,096 (**Supplementary Table S3**). Our transcriptome assembly results are consistent with those of previous studies, which generally showed more genes in conifers than in diploid angiosperms (Mosca et al., 2019; Niu et al., 2022). The contig N50 of the species ranged from 1438 bp to 1882 bp based on all transcript contigs, while the N50s (1292 bp -1767 bp) based on the longest transcript per gene were smaller (**Supplementary Table S3**).

Based on 1614 BUSCOs (embryophyta_odb10 database), we assessed the annotation completeness of the transcriptomes of 18 species with the transcriptome and protein modes. The BUSCO results showed that the assembly integrity of the vast majority of transcriptomes was greater than 80% in both transcriptome mode and protein mode (**Supplementary Tables S4**, **5**). Only two transcriptomes had an assembly integrity value less than 80% in transcriptome mode and protein mode, and this occurred with the common species *Larix gmelinii*.

**Figure 1:**
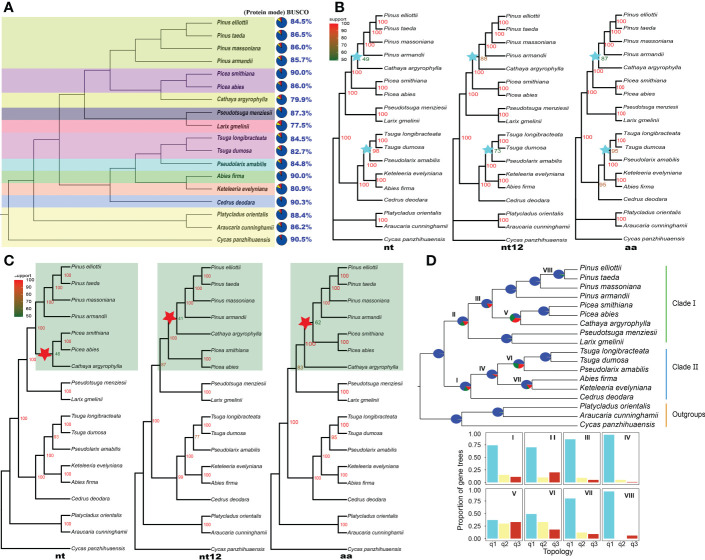
Topology and gene tree conflict analysis of Pinaceae. **(A)** The assessment of annotation completeness based on 1614 benchmarking universal single-copy orthologs (BUSCOs) using protein mode. The colors of the pies refer to complete BUSCOs (steel blue), fragmented BUSCOs (yellow), and missing BUSCOs (red). **(B)** Concatenated ML trees inferred based on 54 single-copy orthologous genes; nt, nucleotide sequences; nt12, 1st + 2nd codon positions; aa, amino acid sequences. IQ-TREE maximum likelihood bootstrap support values are indicated above the branches. **(C)** ASTRAL coalescent trees inferred based on 54 single-copy orthologous genes of 18 species; nt, nucleotide sequences; nt12, 1st + 2nd codon positions; aa, amino acid sequences. Posterior probabilities of the ASTRAL coalescent analyses are indicated above the nodes. **(D)** PHYPARTS coalescent tree analysis. A coalescent tree was constructed from the nt sequence dataset of 54 single-copy orthologous genes using ASTRAL. I, II, III, VI, V, VI, VII and VIII indicate internal branches for which the pie charts depict gene tree incongruence. The colors of pies indicate the following: Support the shown topology (Blue); Conflict with the shown topology (most common conflicting bipartition) (Green); Conflict with the shown topology (all other supported conflicting bipartitions) (Red); and No support for the conflicting bipartition (Gray). The proportion of gene trees was calculated for the three possible arrangements (q1 to q3) for the respective internal branches. The histograms showed quartet support for the main topology (q1), the first alternative topology (q2) and the second alternative topology (q3).

BUSCOs covered 92.7% (**Supplementary Table S5**) of the 1614 core genes and 86.5% of the complete genes in the loblolly pine transcriptome in protein mode (**Figure 1A**). The transcriptome integrity of slash pine and Masson pine was 84.5% and 86.0%, respectively. In addition, the transcriptome integrity of 5 species reached 90%. These results demonstrate the high precision and integrity of the assembled transcriptomes, providing confidence for subsequent phylogenetic and other analyses.

### Instability of concatenated trees

Fifty-four SCOGs were identified using OrthoFinder Version 2.5.4 (Emms and Kelly, 2019) from the transcriptomes of 18 plants. Based on the nt, nt12 and aa sequences of these SCOGs, concatenated ML trees (**Figure 1B**) were inferred using IQ-TREE 2 software (Minh et al., 2020).

The topologies of three concatenated ML trees from nt, nt12, and aa sequences were consistent, but the ML bootstrap support values on some of the same branches were different. The bootstrap value of the clades formed by *Pinus* species (*P. taeda*, *P. massoniana* and *P. armandii*) and the *C. argyrophylla* was <50 in the nt-based ML tree (**Figure 1B**), indicating that the topology was unstable and that a potential alternative topology needs to be considered. The bootstrap values of the clades consisting of *Tsuga* species (*T. dumosa* and *T. longibracteata*) and *P. amabilis* were also quite different among the three ML trees. The bootstrap value of these clade was 73 on the nt12 ML tree, and on the other two ML trees, it was greater than 95 (**Figure 1B**). The Pinaceae topologies of the three concatenated ML trees based on 16 species’ 120 SCOGs (**Supplementary Figure S1**) were consistent with the results from 18 species’ 54 SCOGs.

### Topology conflicts of coalescent trees

To understand the topology reliability of ASTRAL coalescent trees, the 54 SCOG dataset and ASTRAL software (Zhang et al., 2018) were used to infer the coalescent trees, which produced topological trees different from those for the nt, nt12 and aa sequences (**Figure 1C**). The topology conflicts mainly manifested in clades composed of *Pinus* species (*P. elliottii*, *P. taeda*, *P. massoniana*, and *P. armandii*), *Picea* species (*P. abies* and *P. smithiana*), and *C. argyrophylla* (**Figure 1C**, highlighted area). In the nt tree, the *Cathaya* species and *Picea* species are sister groups. However, the sister relationship between *Cathaya* and *Pinus* was closer than that between *Cathaya* and *Picea* from the nt12 tree. Additionally, in the aa tree, *Pinus* species and *Picea* species formed a monophyletic group. Furthermore, the changing branches in the conflicting topologies showed low posterior probability support values (41-62; **Figure 1C**, red star). In ASTRAL coalescent trees of 16 species’ 120 SCOGs (**Supplementary Figure S2**), topologies composed of *Pinus* species, *Picea* species, and the *Cathaya* plant were consistent. However, there were conflicting topologies formed by *Tsuga* plants *(T. dumosa* and *T. longibracteata*), *P. amabilis*, *A. firma*, and *K. evelyniana*. The topology of the nt12 tree (**Supplementary Figure S2B**) was the same as that of the aa tree (**Supplementary Figure S2C**), but it was inconsistent with that of the nt tree (**Supplementary Figure S2A**).

### Conflicts of coalescent gene trees

Based on the nt sequences of the 54 SCOGs, PHYPARTS software (Smith et al., 2015) was used to infer and display gene tree conflicts. PHYPARTS analysis shows that although gene tree topologies and the species topology were concordant at more than half of the nodes, many gene tree topologies conflicted with a given species topology at some nodes (**Figure 1D**). The support level to expect from the gene trees varied greatly at different nodes. At internal branches I and II, the gene tree support for the species topology was less than 75%, meaning that there was a dominant alternative topology that should be considered. The quartet scores indicated that many gene trees supported the first alternative topology (q2) and the second alternative topology (q3), except for the main topology (q1) on branches V and VI. The gene tree conflicts were more pronounced on coalescent trees for nt12 and aa sequences (**Supplementary Figure S3**). Similarly, the topology of the ASTRAL coalescent trees constructed based on the 120 SCOGs also conflicted with the individual gene trees from the nt, nt12 and aa sequences (**Supplementary Figure S4**).

### Ultimate species tree and divergence time

**Figure 2:**
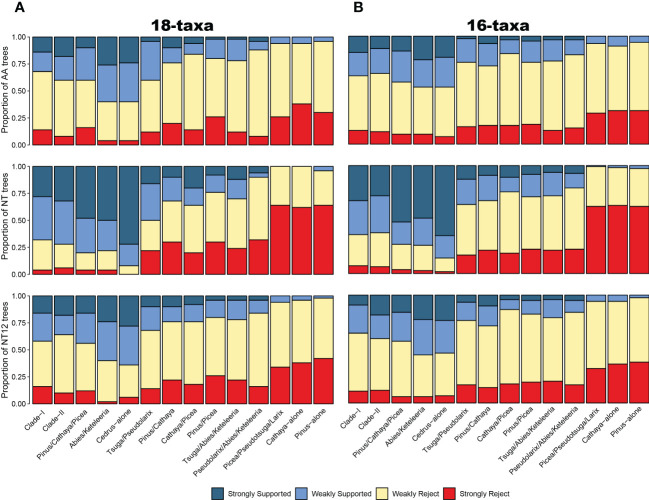
Gene tree analysis. The portion of ML gene trees for which important clades (x-axis) are highly (weakly) supported or rejected for the 54 single-copy orthologous gene dataset **(A)** and 120 single-copy orthologous gene dataset **(B)** and for AA datasets (upper), nucleotide datasets (middle), and 1st+2nd codon position datasets (below). AA: amino acid sequences; NT: nucleotide sequences; NT12: 1st+2nd codon positions. Weakly rejected clades are those that are not in the tree but are compatible if low-support branches (below 75%) are collapsed.

To account for the consistency and conflict of gene trees and topologies among different reconstruction methods and datasets, we used DISCOVISTA software (Sayyari et al., 2018) to summarize gene trees and the inferred 18 species trees. Based on the previous analysis, topological conflicts mainly occurred in two internal clades: 1 clades composed of *Pinus*, *Picea*, and *Cathaya* (**Figure 1C**) and 2) clades composed of *Tsuga*, *Pseudolarix*, *Abies* and *Keteleeria* (**Supplementary Figure S2**). Regarding the several focal phylogenetic relationships from the ASTRAL tree (**Figure 1D**), we generated 14 putative test groups and inspected their monophyly in detail: Clade-I, Clade-II, *Pinus*/*Cathay*a/*Picea*, *Abies*/*Keteleeria*, *Cedrus*-alone (*Cedrus*-other Clade-II species), *Tsuga*/*Pseudolarix*, *Pinus*/*Cathaya*, *Cathaya*/*Picea*, *Pinus*/*Picea*, *Tsuga*/*Abies*/*Keteleeria*, *Pseudolarix*/*Abies*/*Keteleeria*, *Picea*/*Pseudotsuga*/*Larix*, *Cathaya*-alone (Cathaya-other Clade-I species), and *Pinus*-alone (Pinus-other Clade-I species). We observed strong gene tree discordance between individual gene trees in the clustering of the Clade I and Clade II plant groups. Gene tree analyses of 54 SCOG datasets showed that the sister relationship between *Cathaya* and *Picea* obtained the highest level of gene tree support, followed by that of *Cathaya* and *Pinus*, while that of *Pinus* and *Picea* obtained the least support (**Figure 2A**). Instead, the results of 120 SCOG datasets showed that the sister relationship between *Cathaya* and *Pinus* had the highest average level of gene tree support, followed by that of *Cathaya* and *Picea*, whereas the monophyletic relationship of *Pinus* and *Picea* obtained the least support (**Figure 2B**).

**Figure 3:**
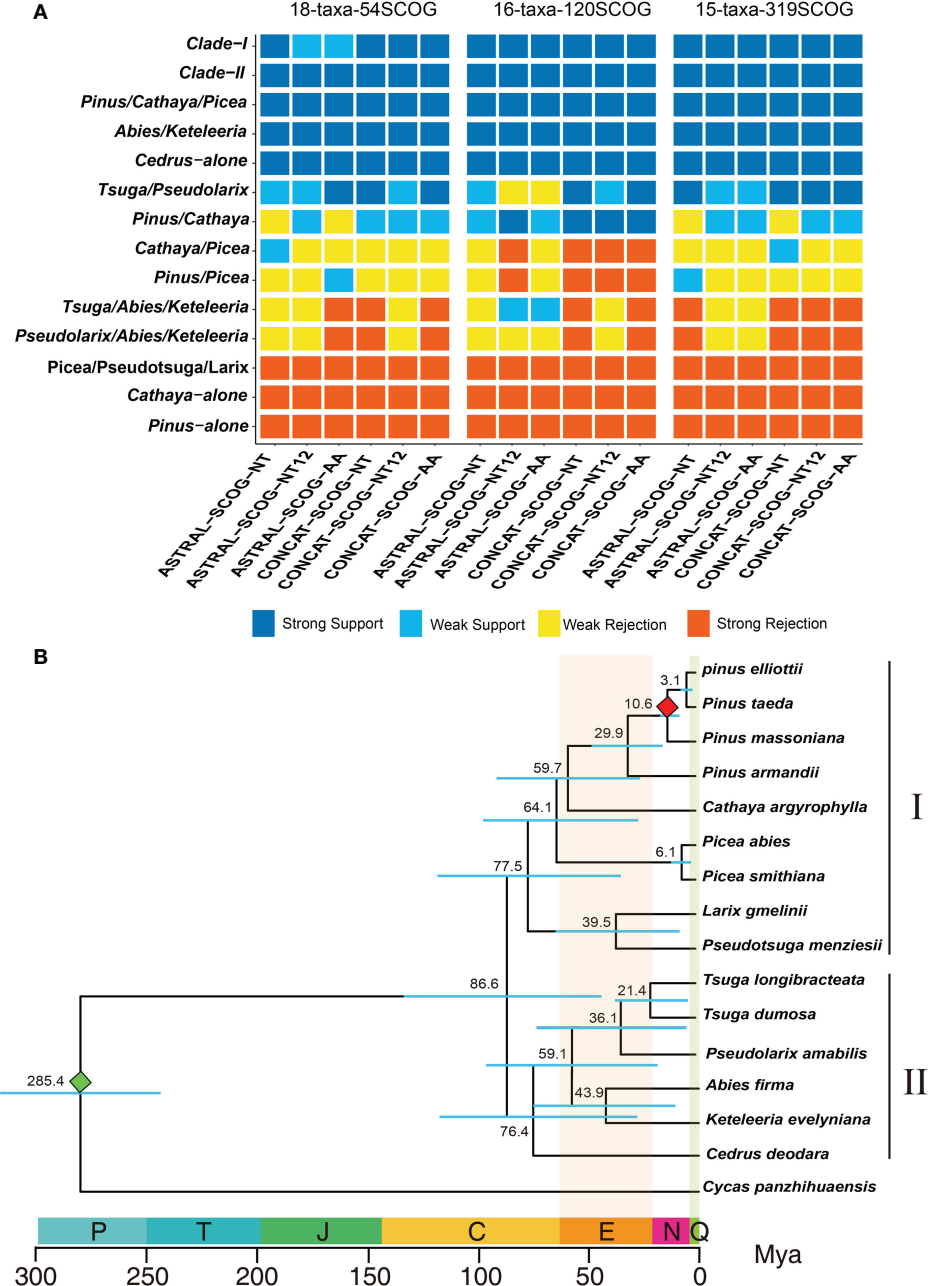
Determination of the final species tree and divergence time calculation. **(A)** DiscoVista species tree analysis: rows correspond to the 14 hypothetical groups tested, and columns correspond to the results derived from the use of different datasets and methods. SCOG, single-copy orthologous gene; NT, nucleotide sequences; NT12, 1st + 2nd codon positions; AA, amino acid sequences; ASTRAL, coalescent tree inference method using ASTRAL; CONCAT, maximum likelihood tree inferred with IQ-TREE based on concatenated datasets. Strong support, the clade is reconstructed with a support value >95%. Weak support, the clade is reconstructed with a support value <95%. Weak rejection, the clade is not recovered, but the alternative topology is not conflicting if poorly supported branches (<85%) are collapsed. Strong rejection, the clade is not recovered, and the alternative topology conflicts even when poorly supported branches (<85%) are collapsed. **(B)** Chronogram of pine plants on the basis of 120 single-copy orthologous genes’ 1st+2nd codon sequences inferred using MCMCTree. The red and green diamonds indicate the divergence time of *P. massoniana* and *C. panzhihuaensis*, respectively. P, Permian; T, Triassic; J, Jurassic; C, Cretaceous; N, Neogene; Q, Quaternary; Mya, million years ago.

Both the coalescent species tree inferred by ASTRAL and the best ML tree inferred from concatenated datasets consistently and strongly supported the division of the 10 genera of Pinaceae into two major groups: Clade-I and Clade-II (**Figure 3A**). In Clade-I, the monophyletic analyses strongly supported that *Pinus*, *Cathaya*, and *Picea* (*Pinus/Cathaya/Picea*) are from a common ancestor. Additionally, analyses strongly rejected sister relationships between *Pinus* and *Picea* and between *Cathaya* and *Picea* (4 species trees showed strong rejection, 12 species trees showed weak rejection, and 2 species trees showed weak support), whereas comparisons supported *Pinus/Cathaya* as a sister clade (4 species trees showed weak rejection, 10 species trees showed weak support, and 4 species trees showed strong support) (**Figure 3A**). In Clade-II, both the phylogenetic relationship of *Cedrus* alone and the sister relationship of *Abies* and *Keteleeria* (*Abies*/*Keteleeria*) were strongly supported by 18 species trees. The sister relationships between *Pseudolarix* and *Abies*+*Keteleeria* (*Pseudolarix/Abies/Keteleeria*) and between *Tsuga* and *Abies*+*Keteleeria* (*Tsuga/Abies/Keteleeria*) were rejected strongly by 9 trees and weakly by 9 trees (**Figure 3A**). On the other hand, the monophyletic group of *Tsuga* and *Pseudolarix* was strongly supported by 9 species trees and weakly supported by 9 species trees (**Figure 3A**). Therefore, the most likely phylogenetic tree of Pinaceae is ((((*Pinus*, *Cathaya*), *Picea*), *Pseudotsuga*, *Larix*), (((*Tsuga*, *Pseudolarix*), (*Abies*, *Keteleeria*)), *Cedarus*)).

Using the final Pinaceae phylogenetic tree, we estimated divergence times for 16 species. We estimated the divergence times of species based on the 1^st^+2^nd^ codon sequences of 120 SCOGs. Fifteen species of 10 genera of Pinaceae could be divided into clade I and clade II, which diverge 86.6 million years ago (Mya) (**Figure 3B**). Clade I includes 5 genera and 9 species, may have had a common ancestor in the Cretaceous period 77.5 Mya. Clade II includes 5 genera, may have had a common ancestor 76.4 Mya in the Cretaceous period. Clade I was estimated to have diverged from Clade II around 86.6 million years ago. These results are consistent with the view of Cretaceous radiation of Pinaceae species (White et al., 2007). The dated phylogenetic tree shows that *P. taeda* likely diverged from *P. elliottii*, *P. massoniana* and *P. armandii* approximately 3.1, 10.6 and 29.9 Mya, respectively. Although Pinaceae has an ancient origin, some existing species of *Pinus* have not diverged for a long time and are very young (Gernandt et al., 2008; Hao et al., 2015; Shao et al., 2019; Shen et al., 2019).

### Expansion and contraction of gene families

**Figure 4:**
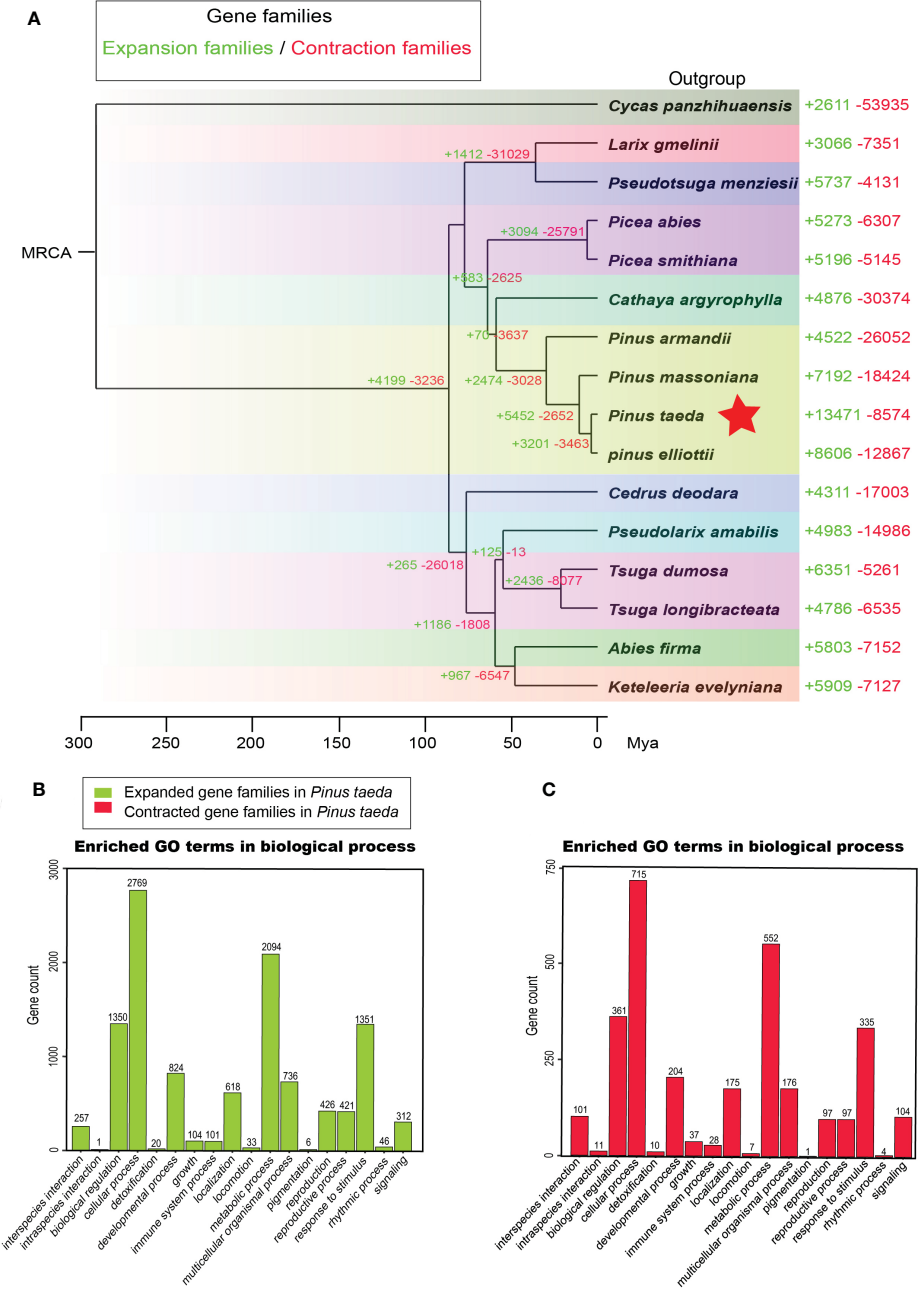
Gene family analysis. **(A)** The evolution of gene families in 15 species of Pinaceae and one outgroup (Cycadaceae: *Cycas panzhihuaensis*). The corresponding numbers indicate gain (expansion) or loss (contraction) of gene families in specific species. **(B)** Gene Ontology (GO) enrichment analysis of expanded gene families in *Pinus taeda*. **(C)** Gene Ontology (GO) enrichment analysis of contracted gene families in *Pinus taeda*.

We examined the sizes of expanded and contracted gene families in 15 Pinaceae species representing 10 genera: *A. firma*, *C. argyrophylla*, *C. deodara*, *K. evelyniana*, *L. gmelinii*, *P. abies*, *P. smithiana*, *P.s armandii*, *P. elliottii*, *P. massoniana*, *P. taeda*, *P. amabilis*, *P. menziesii*, *T. dumosa* and *T. longibracteata*. We identified 13471 expanded and 8574 contracted gene families in *P. taeda* (**Figure 4A**). Compared to that in other plant transcriptomes, the number of expanded gene families was the largest in *P. taeda*. Notably, the significantly expanded gene families in *P. taeda* were mainly related to biological regulation, cellular processes, metabolic processes, and responses to stimuli in the “biological process” category of GO analysis (**Figure 4B**; **Supplementary Figure S5**, **Table S6**). Although the significantly contracted gene families in *P. taeda* were also mainly associated with these “biological process” genes, they were much less common than the expanded gene families (**Figure 4C**, **Supplementary Figure S6**; **Table S7**).

KEGG annotation further confirmed the expansion of metabolic genes in *P. taeda* (**Supplementary Figure S7**). The expanded and contracted gene families were mainly enriched in KEGG terms such as BRITE hierarchies and metabolism, which included carbohydrate metabolism, metabolism of cofactors and vitamins, amino acid metabolism, metabolism of terpenoids and polyketides, xenobiotic biodegradation and metabolism, and biosynthesis of other secondary metabolites (**Supplementary Figure S7**, **S8**). Notably, there was a specific class of BRITE hierarchies (protein families: metabolism) associated with the expanded gene families compared with the contracted gene families.

### Evolution of terpene biosynthesis

The stems and leaves of conifers synthesize and secrete oleoresin, which contains a variety of terpenoid compounds as an induced defense response against attacks by herbivores, insects, and pathogens (Keeling and Bohlmann, 2006; Liu et al., 2021). Moreover, terpenoid metabolism plays a crucial role in adapting to environmental conditions (Celedon and Bohlmann, 2019). In conifers, all terpenoid metabolic compounds are derived from two pathways: the chloroplast methyl erythritol phosphate (MEP) and cytosolic mevalonate (MEV) pathways (Celedon and Bohlmann, 2019; Niu et al., 2022). In these two pathways, TPSs and the P450 superfamily are key catalytic enzyme genes, which control the final step of resin terpene biosynthesis (Celedon and Bohlmann, 2019; Niu et al., 2022).

**Figure 5:**
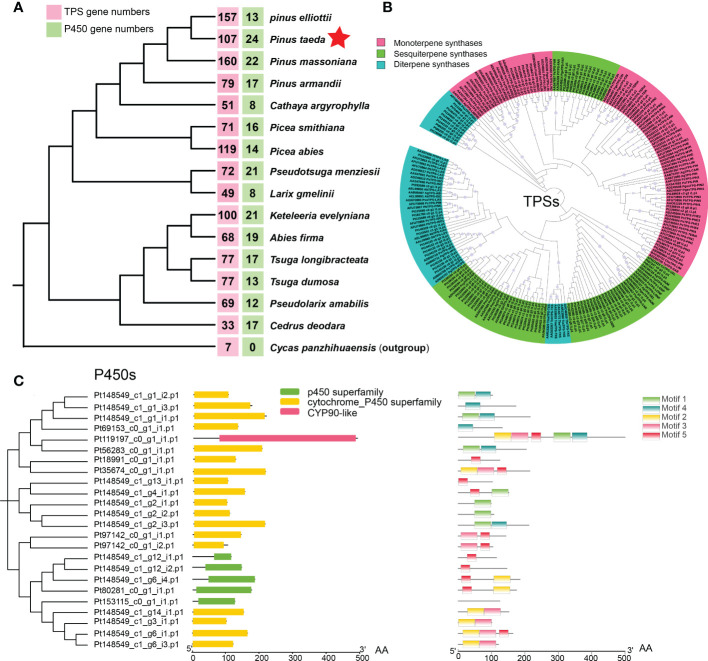
Evolution of the coniferous terpene synthase (TPS) family and P450 family. **(A)** The numbers of TPS genes (red box) and P450 genes (green box) in 16 gymnosperms (15 Pinaceae plants and 1 outgroup). **(B)** Maximum likelihood phylogenetic tree of terpene synthases (TPSs) in conifers. **(C)** Domain and motif of P450s in *Pinus taeda* amino acid sequences.

To investigate the evolution of terpene biosynthesis, 111 candidate genes encoding TPSs and 23 candidate genes encoding P450 proteins were analyzed for homologs in different species (**Figure 5A**). Different conifer species have similar numbers of catalytic enzyme genes for most steps in the MEP pathway and MEV pathway (Celedon and Bohlmann, 2019; Niu et al., 2022). However, the number of key enzymes of TPSs and P450s differs greatly among species. One example is the number of TPS gene families in different gymnosperms. Compared with that in the ancestral gymnosperm (*Cycad panzhihuaensis*), the conifer TPS family, including monoterpene synthases, sesquiterpene synthases, and diterpene synthase, has expanded to 160 genes in *P. massoniana* (**Figure 5A**), much higher than in any other conifers (Niu et al., 2022; Sun et al., 2022). *P. taeda* has 107 TPS genes, including 26 monoterpene synthases, 57 sesquiterpene synthases, and 24 diterpene synthases (**Figure 5B**). Another example is the number of P450 gene families in different conifers. In contrast with eight P450 genes in *L. gmelinii*, *P. taeda* has 24 P450 genes. The 24 potential P450 genes in *P. taeda* include genes in the P450 superfamily, cytochrome p450 superfamily and CYP90-like family according to their domains (**Figure 5C**). The CYP90-like family is composed of plant cytochrome P450s that catalyze the oxidative 5,6-spiroketalization of cholesterol to produce diosgenin (Christ et al., 2019), which is a recently discovered defense compound in plants.

To further elucidate the evolution of the TPS and P450 families in *P. taeda*, conifer genes encoding TPSs and P450s were used to search for homologs among genes with significant expansion and contraction. We found three TPS genes among the significantly contracted gene families of *P. taeda* (**Supplementary Figure S9A**). However, no TPS genes were found among the significantly expanded gene families. In contrast, we found 11 P450 genes among the expanded gene families (**Supplementary Figure S9B**) and no P450 genes among the contracted gene families. This is probably why *P. taeda* has the fewest TPS genes and the most P450 genes among the young *Pinus* species (*P. massoniana*, *P. elliottii*, and *P. taeda*).

### Expression patterns of TPS and P450 genes

**Figure 6:**
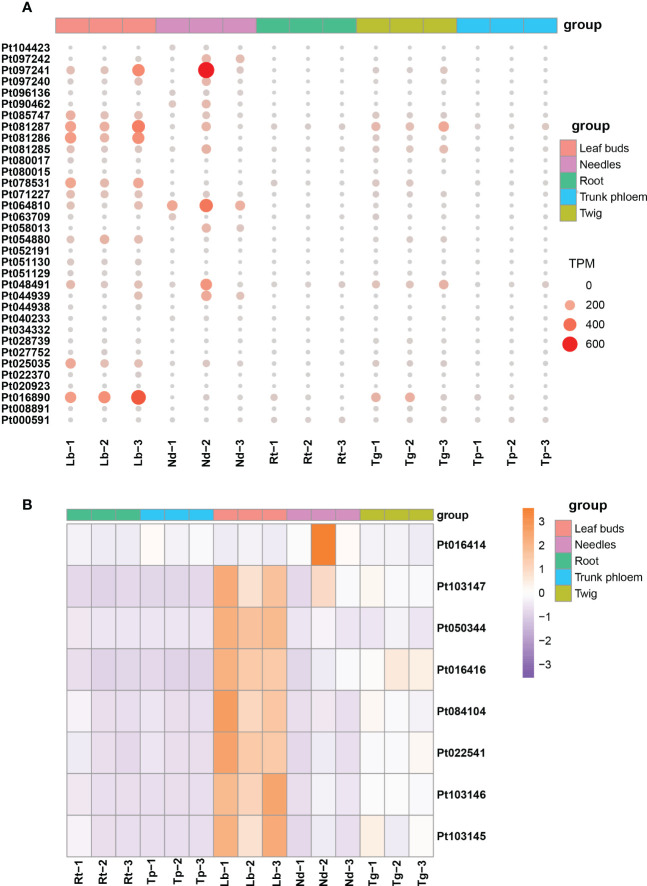
The expression profiles for TPS and P450 genes in different tissues of *Pinus taeda*. **(A)** Expression levels of TPS genes in five tissues of *Pinus taeda*. **(B)** The heatmap for P450 genes in different tissues of *Pinus taeda*.

The expression patterns of TPS and P450 protein-related genes were examined in different tissues of loblolly pine. We found that TPS homologs displayed substantially different expression patterns (**Figure 6A**). Most TPS genes were primarily expressed in leaf buds and needles, their expression declined noticeably in twigs, and there was almost no expression in roots and trunk phloem (**Figure 6A**). Similarly, the P450 genes were highly expressed only in leaf buds and needles, with expression gradually decreasing in roots, trunk phloem and twigs (**Figure 6B**). These results are consistent with those of previous research, suggesting that new needles are likely the main tissues for terpenoid biosynthesis (Niu et al., 2022). In addition, we believe that leaf buds are also one of the main tissues for terpenoid synthesis in conifers.

## Discussion

### Phylogeny of Pinaceae

In the latest classification system, Pinaceae includes 11 genera: *Pinus*, *Pseudotsuga*, *Picea*, *Larix*, *Abies*, Nothotsuga, *Cathaya*, *Cedrus*, *Keteleeria*, *Pseudolarix*, and *Tsuga*. Originally, Pinaceae species were usually classified on the basis of morphological characteristics, mainly those of leaves, seeds, and cones (Shaw, 1914). In the early stage, the phylogeny of Pinaceae was constructed based on morphology (Hart, 1987; Liston et al., 2003), immunology (Price et al., 1987) and a few genes (Gernandt et al., 2005). In the last decade, to determine the relationships between the genera of Pinaceae, researchers have conducted phylogenetic studies using more genes (Gernandt et al., 2016; Ran et al., 2018). Single-copy orthologous genes are more suitable for constructing phylogenetic trees. Here, we used different datasets and different inference methods to infer species trees for 15 species in 10 genera of Pinaceae with transcriptomes. From the analysis of the inferred 18-species trees, the most likely topology of Pinaceae was (*Cycas*, ((((*Pinus*, *Cathaya*), *Picea*), *Pseudotsuga*, *Larix*), (((*Tsuga*, *Pseudolarix*), (*Abies*, *Keteleeria*)), *Cedarus*))), with *Cycas* as an outgroup. This result is consistent with previous research (Gernandt et al., 2016; Ran et al., 2018).

Fossils suggested that gymnosperms first appeared during the Devonian (409 Mya) or Carboniferous (363 Mya) period (Niklas, 1997). Subsequently (approximately 225 Mya), conifers flourished, dominating the flora and rapidly radiating (White et al., 2007). Pinaceae, a family of modern conifers, evolved during the Triassic and Jurassic periods and underwent radiative evolution during the Cretaceous (Miller, 1976). The warm and dry climate of the Cretaceous period (65-136 Mya) favored the expansion of Pinaceae trees in the northern mid-latitudes (Millar, 1998; Eckert and Hall, 2006; Jin et al., 2021). Our dated phylogenetic tree shows that the two major clades of Pinaceae diverged during the Cretaceous period, suggesting that Pinaceae evolution generated a great deal of diversity in this period.

### Evolution of gene families in Pinaceae

Conifers have extremely large genomes, especially Pinaceae, which have greater genome sizes than other gymnosperms (Murray, 1998). Compared with angiosperms (El Baidouri and Panaud, 2013; Cossu et al., 2017), pines have a longer half-life of transposable elements (Nystedt et al., 2013) and a lower removal rate of long terminal repeats (Niu et al., 2022), resulting in large genomes composed of repetitive sequences (Niu et al., 2022). In this study, we observed a large number of expanded gene families in the family Pinaceae, especially in the genus *Pinus*, with loblolly pine having the most expanded gene families (**Figure 4**). One possible explanation is that these gene families actually expand through transposable elements.

Terpenoid metabolism is a protective mechanism of pines, which can not only respond to disease and insect damage but also respond to environmental stress (Celedon and Bohlmann, 2019; Zhao and Erbilgin, 2019; Liu et al., 2021). Conifers release considerable amounts of volatile terpenes, which may contribute to the possible interplant signaling of stress stimuli. Differences in the quantity and quality of terpenes emission may be a sign of the tree adaptations to the changing environment and the pressure exerted by stress factors (Kopaczyk et al., 2020). In loblolly pine, expanded genes were mainly enriched in GO terms related to biological regulation, cellular processes, metabolic processes, and responses to stimulus, indicating that increasing terpenoids in response to environmental stress is the main evolutionary direction. In addition, TPS and P450 proteins are key rate-limiting enzymes in the last two steps of the two pathways of terpene biosynthesis (Celedon and Bohlmann, 2019; Mao et al., 2019; Niu et al., 2022); we found a decrease in the TPS family and an increase in the P450 family in loblolly pine. Consistent with the results of previous studies (Niu et al., 2022), TPS and P450 family genes were mainly expressed in needles and leaf buds, and their expression decreased rapidly in roots, trunk phloem, and twigs. One intriguing explanation is that leaf buds and needles are the tissues where the genes for terpene synthesis have been most active over millions of years of evolution.

Overall, the application of different datasets and multiple species tree inference methods provided insights into the complex Pinaceae phylogeny. Gene family analyses of TPSs and P450s improved our understanding of the evolution of terpene biosynthesis in conifers.

## Limitations of the study

Nothotsuga is a monotypic genus of Pinaceae endemic to southern China today. Nothotsuga was not included in the study, which was not consummate. However, the topology of the phylogeny of the Pinaceae was consistent with previous reports (Gernandt et al., 2016; Ran et al., 2018). As the sister group of Tsuga (Ran et al., 2018), Nothotsuga split from Tsuga during the Paleogene (Ding et al., 2021).

## Data availability statement

The datasets presented in this study can be found in online repositories. The names of the repository/repositories and accession number(s) can be found in the article/**Supplementary Material**.

## Author contributions

SH and TL conceived the project. KJ participated in study design and coordination, performed the lab work, processed the experimental data, interpreted the data, and drafted the manuscript. CD and LH performed the lab work and helped in sampling. KJ and JL conducted the analyses. SH, TL and KJ revised the manuscript. All authors contributed to the article and approved the submitted version.
