# Supplementary material for: Phylotranscriptomics and evolution of key genes for terpene biosynthesis in Pinaceae
Source: Front Plant Sci. 2023 Feb 17;14:1114579. doi: 10.3389/fpls.2023.1114579 (PMC9982022; doi:10.3389/fpls.2023.1114579)
Supplement: Supplementary file 1 [file DataSheet_1.docx]

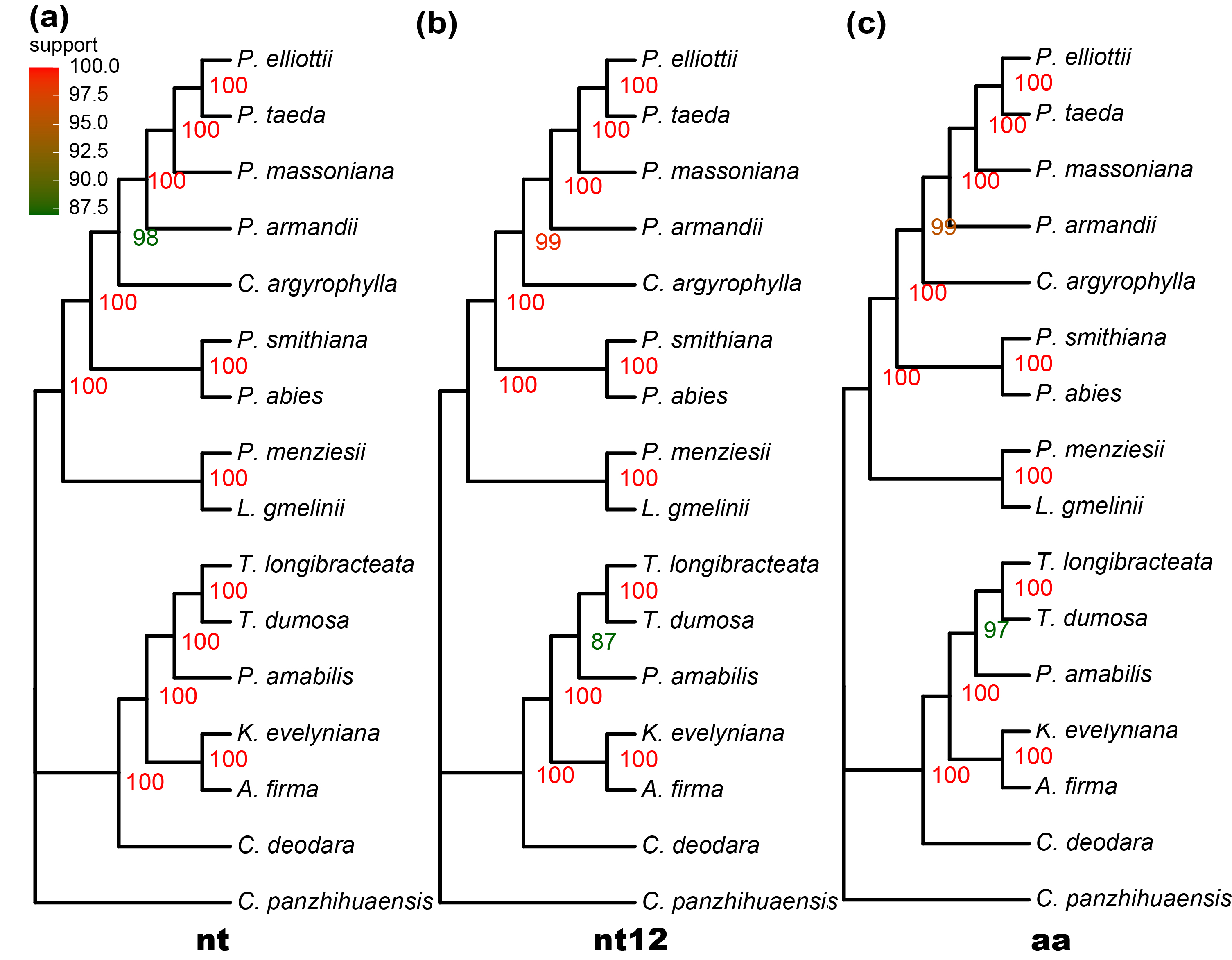


**Fig. S1** Concatenated ML tree inferred based on 120 single-copy orthologous genes. ML trees was inferred based on the codon (nt) sequence (a), codon 1+2 (nt12) sequence (b), and amino acid (aa) sequence (c) of 120 coding genes, respectively. IQTREE maximum likelihood bootstrap support values were indicated above the branches. Scientific name of 16 species are *Abies firma*, *Cathaya argyrophylla*, *Cedrus deodara*, *Cycas panzhihuaensis*, *Keteleeria evelyniana*, *Larix gmelinii*, *Picea abies*, *Picea smithiana*, *Pinus armandii*, *Pinus elliottii*, *Pinus massoniana*, *Pinus taeda*, *Pseudolarix amabilis*, *Pseudotsuga menziesii*, *Tsuga Dumosa, Tsuga longibracteata*.


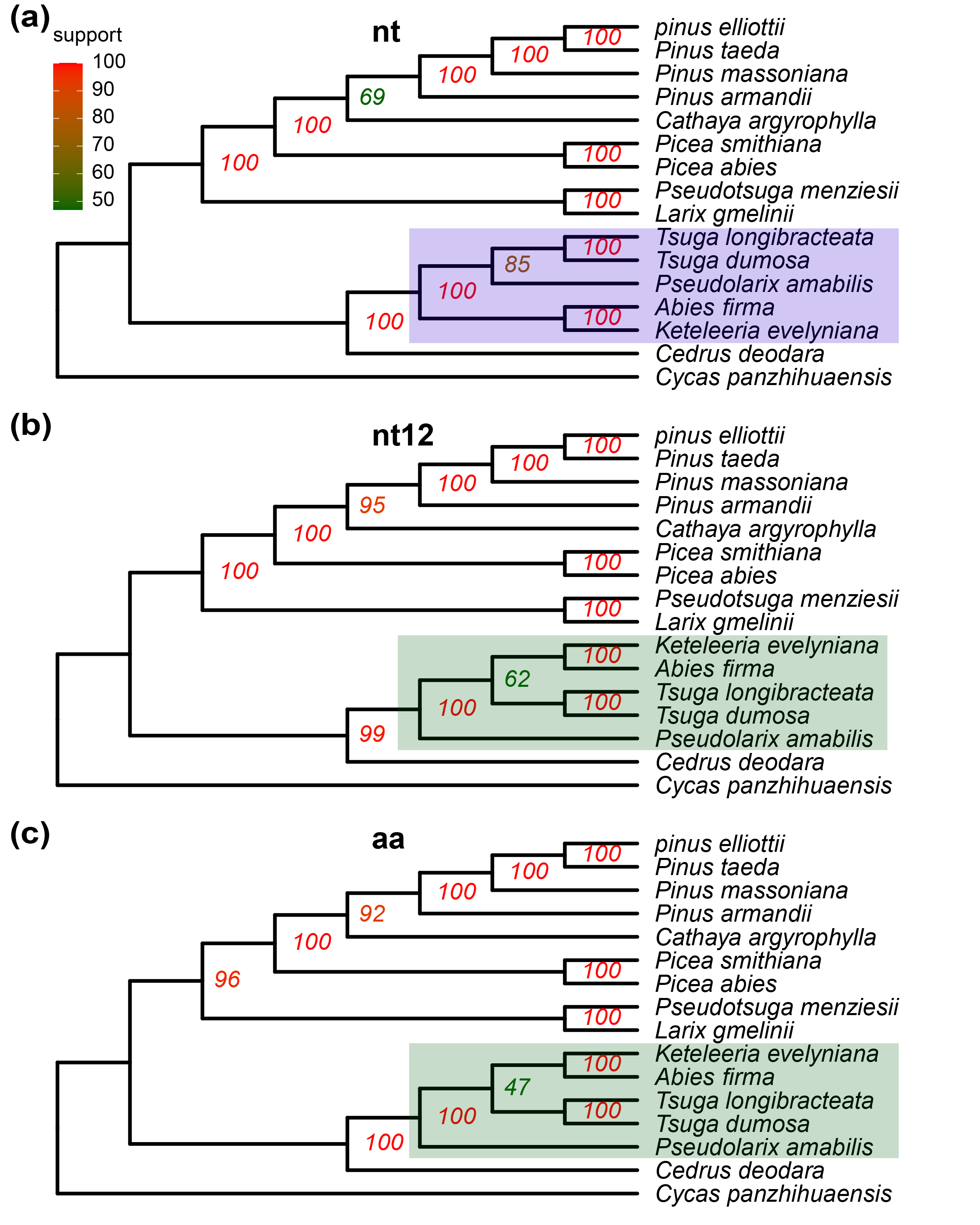


**Fig. S2** ASTRAL coalescent trees inferred based on 120 single-copy orthologous genes of 16 species. ASTRAL coalescent trees were inferred based on codon (nt) sequence (a), codon 1st+2nd (nt12) sequence (b), and amino acid (aa) sequence (c), respectively. Posterior probabilities of the ASTRAL coalescent analyses were indicated above the nodes.


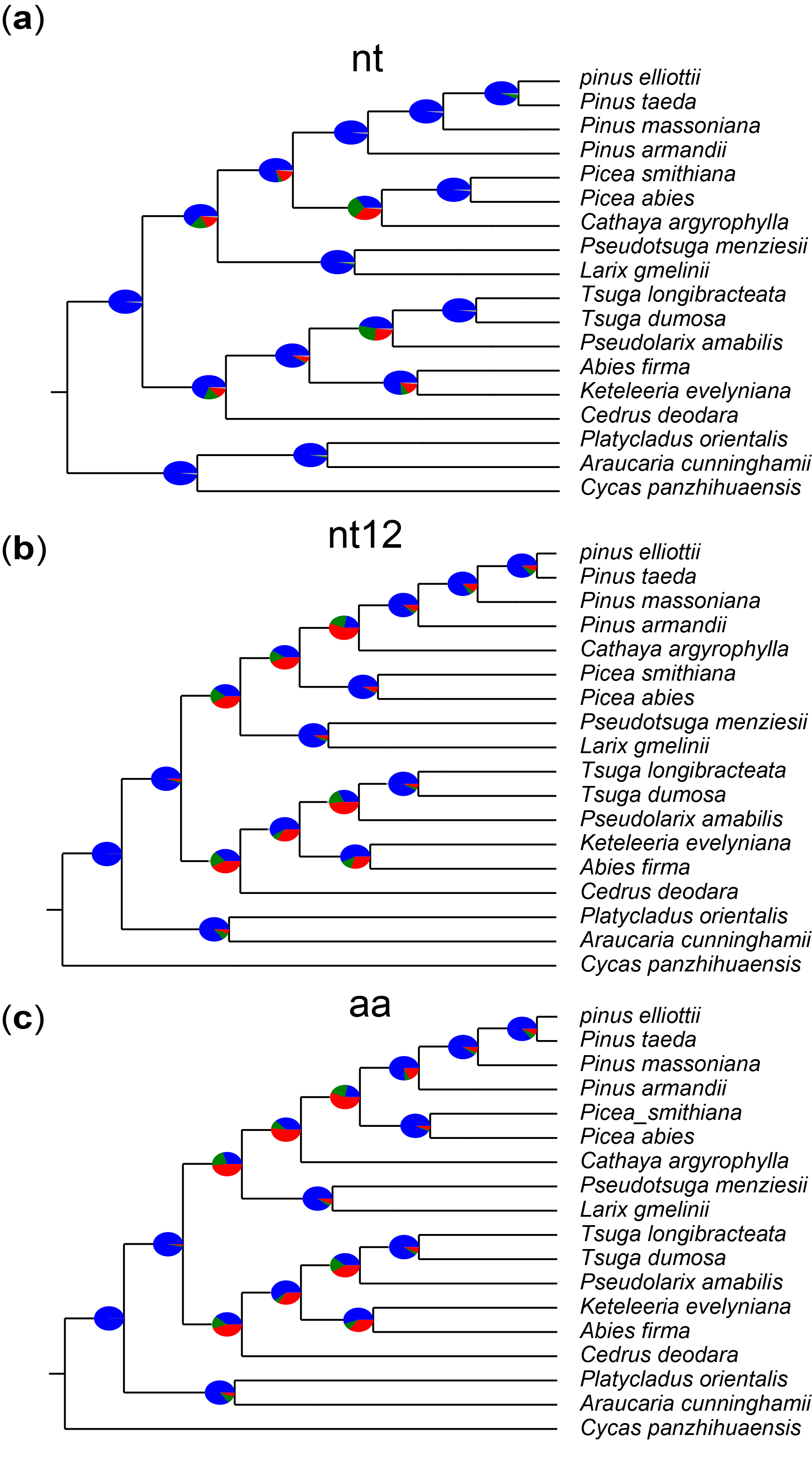


**Fig. S3** PHYPARTS coalescent trees analysis based on 54 single-copy orthologous genes of 18 species. ASTRAL coalescent trees were inferred based on codon (nt) sequence (a), codon 1st+2nd (nt12) sequence (b), and amino acid (aa) sequence (c), respectively. The pie charts on the nodes depicting whether gene tree topologies are concordant or conflicting with the species topologies. The color of pies refer to: Support the shown topology (Blue); Conflict with the shown topology (most common conflicting bipartion) (Green); Conflict with the shown topology (all other supported conflicting bipartitions) (Red); Have no support for conflicting bipartion (Gray).


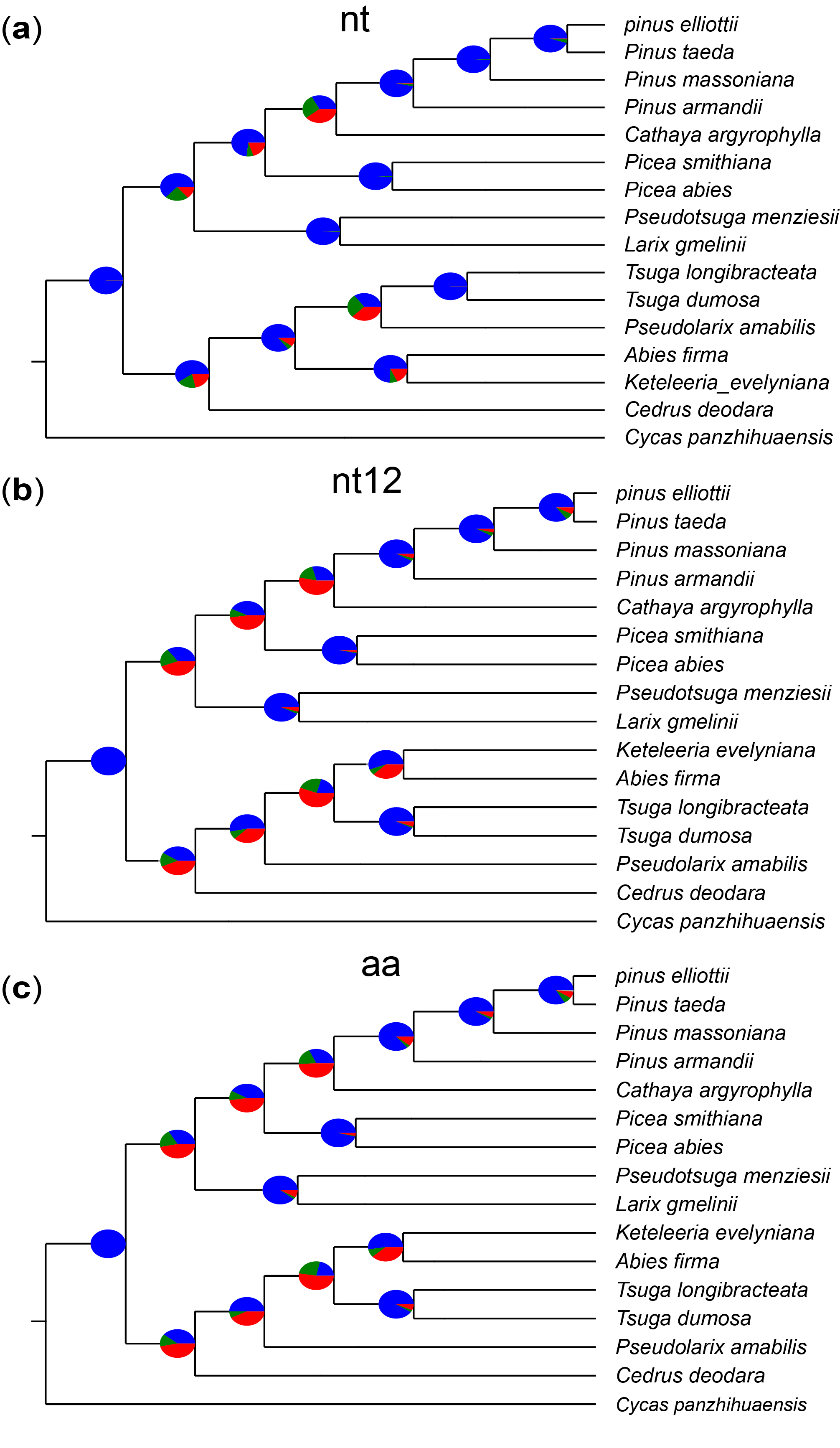


**Fig. S4** PHYPARTS coalescent trees analysis based on 120 single-copy orthologous genes of 16 species. ASTRAL coalescent trees were inferred based on codon (nt) sequence (a), codon 1st+2nd (nt12) sequence (b), and amino acid (aa) sequence (c), respectively. The pie charts on the nodes depicting whether gene tree topologies are concordant or conflicting with the species topologies. The color of pies refer to: Support the shown topology (Blue); Conflict with the shown topology (most common conflicting bipartion) (Green); Conflict with the shown topology (all other supported conflicting bipartitions) (Red); Have no support for conflicting bipartion (Gray).


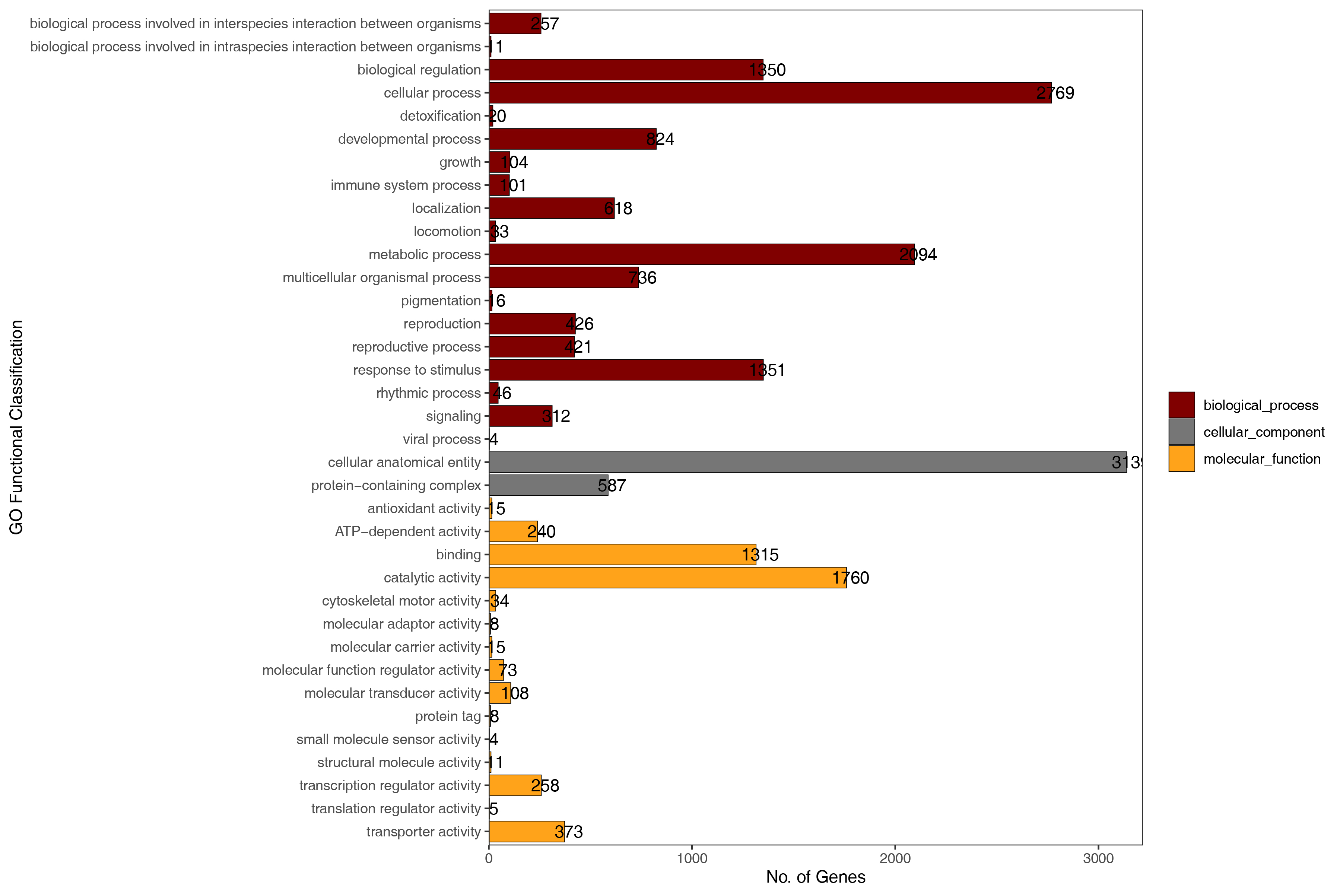


**Fig. S5** GO enrichment analysis of significantly expanded gene families in *Pinus taeda*.


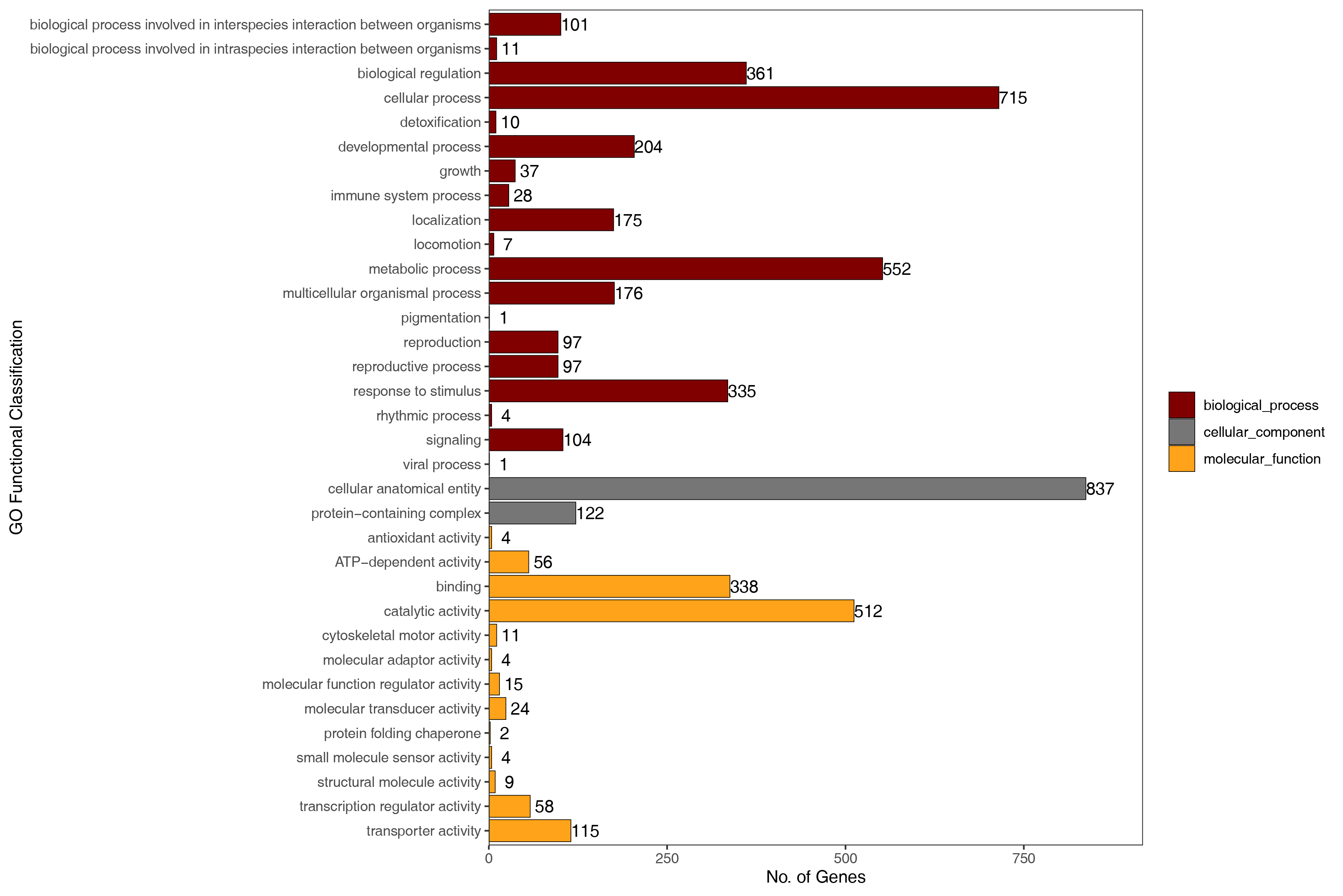


**Fig. S6** GO enrichment analysis of significantly contracted gene families in *Pinus taeda*.


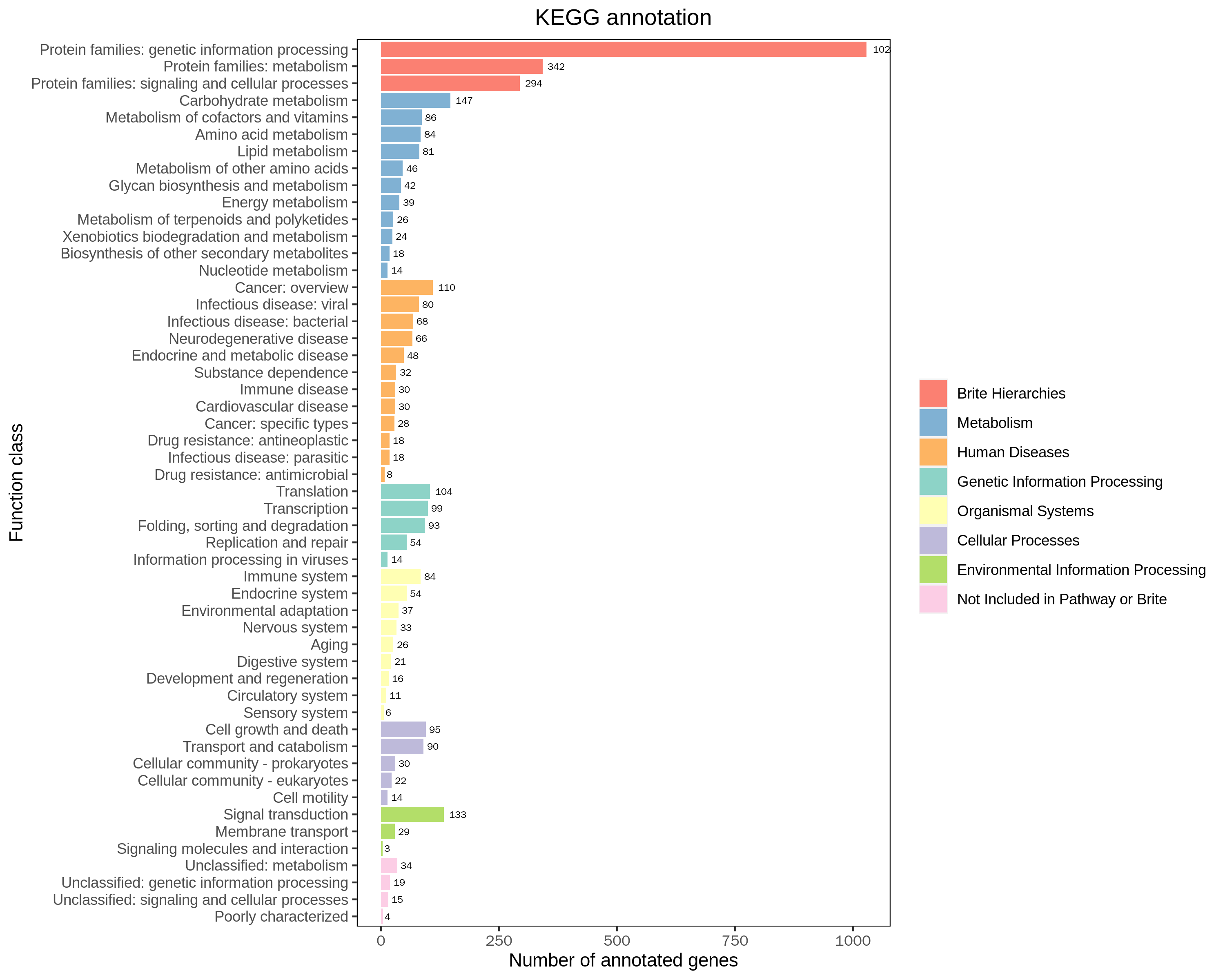


**Fig. S7** KEGG pathway analysis of significantly expansion gene families in *Pinus taeda*.


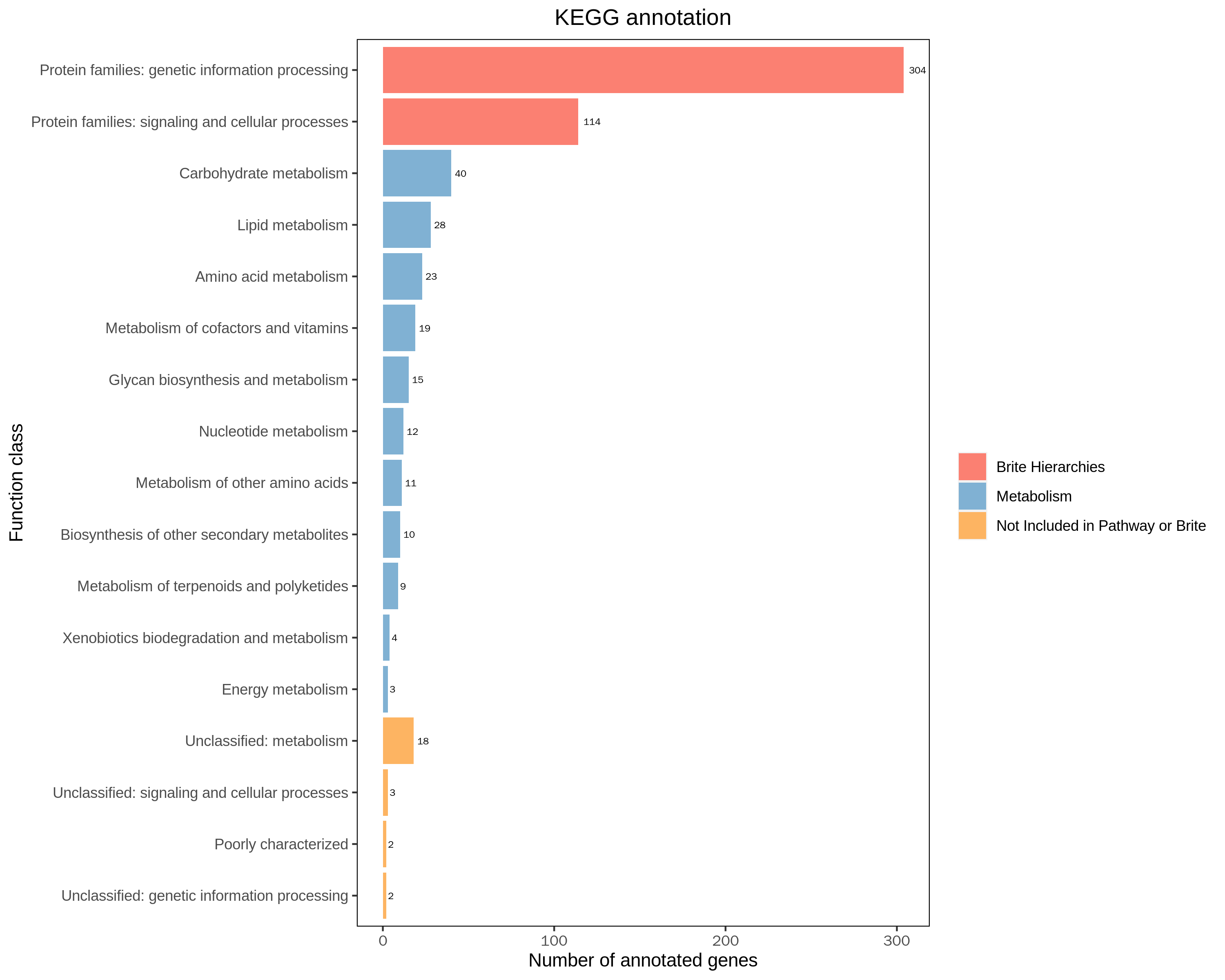


**Fig. S8** KEGG pathway analysis of significantly contraction gene families in *Pinus taeda*.


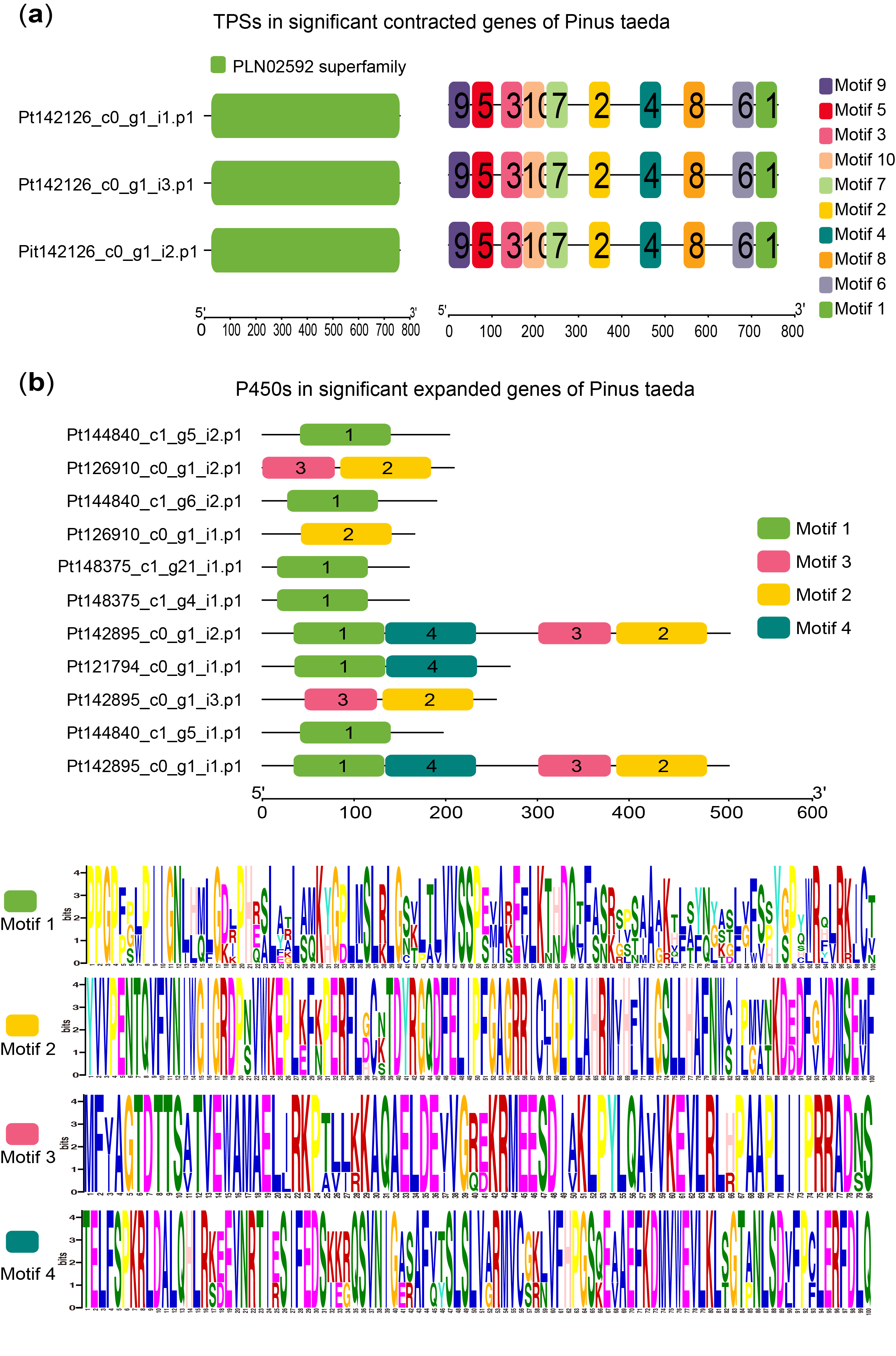


**Fig. S9** Numbers of terpene synthase (TPS) and P450 in expanded and contracted gene families of *Pinus taeda*. (a) TPSs in significant contracted gene family of *Pinus taeda*. (b) P450s in significant expanded gene families of *Pinus taeda*. Notably, No TPS genes were found in significant expanded gene family and there were no P450 genes in significant contracted gene family of *Pinus taeda*.
